# Recent Travel History and *Plasmodium falciparum* Malaria Infection in a Region of Heterogenous Transmission in Southern Province, Zambia

**DOI:** 10.4269/ajtmh.19-0660

**Published:** 2020-07-02

**Authors:** Travis R. Porter, Timothy P. Finn, Kafula Silumbe, Victor Chalwe, Busiku Hamainza, Emmanuel Kooma, Hawela Moonga, Adam Bennett, Joshua O. Yukich, Richard W. Steketee, Joseph Keating, John M. Miller, Thomas P. Eisele

**Affiliations:** 1 Department of Tropical Medicine, Center for Applied Malaria Research and Evaluation, Tulane University School of Public Health and Tropical Medicine, New Orleans, Louisiana;; 2 PATH Malaria Control and Elimination Partnership in Africa (MACEPA), Lusaka, Zambia;; 3 National Malaria Elimination Centre, Zambia Ministry of Health, Lusaka, Zambia;; 4 Malaria Elimination Initiative, Global Health Group, University of California San Francisco, San Francisco, California;; 5 PATH MACEPA, Seattle, Washington

## Abstract

As Zambia continues to reduce its malaria incidence and target elimination in Southern Province, there is a need to identify factors that can reintroduce parasites and sustain malaria transmission. To examine the relative contributions of types of human mobility on malaria prevalence, this analysis quantifies the proportion of the population having recently traveled during both peak and nonpeak transmission seasons over the course of 2 years and assesses the relationship between short-term travel and malaria infection status. Among all residents targeted by mass drug administration in the Lake Kariba region of Southern Province, 602,620 rapid diagnostic tests and recent travel histories were collected during four campaign rounds occurring between December 2014 and February 2016. Rates of short-term travel in the previous 2 weeks fluctuated seasonally from 0.3% to 1.2%. Travel was significantly associated with prevalent malaria infection both seasonally and overall (adjusted odds ratio [AOR]: 2.55; 95% CI: 2.28–2.85). The strength of association between travel and malaria infection varied by travelers’ origin and destination, with those recently traveling to high-prevalence areas from low-prevalence areas experiencing the highest odds of malaria infection (AOR: 7.38). Long-lasting insecticidal net usage while traveling was associated with a relative reduction in infections (AOR: 0.74) compared with travelers not using a net. Although travel was directly associated with only a small fraction of infections, importation of malaria via human movement may play an increasingly important role in this elimination setting as transmission rates continue to decline.

## INTRODUCTION

Scale-up of malaria control and targeted elimination efforts in Southern Province, Zambia, have resulted in demonstrable reductions of at least 60% in malaria incidence and greater than 80% in malaria prevalence since the early 2010s.^1–3^ In the face of recent increases in malaria nationally, broad scale-up of malaria control interventions and gains in lower burden areas has fueled a push for malaria elimination nationwide by 2021.^4^ This has resulted in the adoption of new strategies targeting areas of lower transmission, including the recent implementation of a mass drug administration (MDA) trial throughout the Lake Kariba region of Southern Province.^5^

Insofar as MDA represents a concentrated push toward reducing malaria prevalence, follow-up of previous MDA efforts and modeling studies in this region have suggested that the gains of MDA will diminish over time without further intervention.^6–8^ Maintaining high vector control coverage and scaling up community case management are likely to be important for preventing resurgence in parasite prevalence, maintaining reduced transmission, and continuing progress toward elimination.^8,9^ As malaria control strategies approach the interruption of endemic transmission while reducing the overall malaria burden, preventing reintroduction of parasites via importation from other regions also becomes an increasingly important consideration.^10–12^

Among the risk factors for reintroducing malaria parasites into a population is the circulation of people to and from areas of ongoing transmission.^10,13–15^ Human movement can transmit parasites when residents of malaria-free or low-prevalence areas travel to endemic areas, become infected, and then return to their community of origin, or when infected individuals migrate to or visit these areas from endemic regions.^16^ When an area has adequate vectorial capacity—or is sufficiently receptive—to support endemic transmission, importation can reestablish local transmission. Even still, continuous importation of parasites to areas of low reproductive capacity may perpetually fuel transmission even if the area is incapable of sustaining endemic transmission alone.^14,17,18^

Within sub-Saharan Africa, previous studies have provided empirical evidence demonstrating a general association between travel and individual parasitemia, especially among individuals returning to lower prevalence areas.^19–24^ Not all types of travel pose the same risk for infection nor does travel necessarily translate into a significant threat to low-prevalence areas. An individual’s duration and frequency of travel, risk while traveling, and risk management behaviors contribute to the probability of acquiring parasites.^13,15,25^ More broadly, the frequency of human movement, abundance of high-prevalence areas, and spatial connectedness of regional sources of transmission determine the frequency of higher risk travel and can result in differences in vulnerability between populations.^9,14,26^

Until recently, local transmission intensity in Southern Province has varied substantially between adjacent districts and their health facilities.^5^ Following concentrated malaria control and elimination efforts, many of these health facility catchment areas (HFCAs) now see very low parasite prevalence, although areas of moderate prevalence (> 10% among children younger than 5 years) still remain.^3^ High vector control coverage and expansion of community case management have likely decreased receptivity in this region; however, current low infection prevalence, due in part to punctuated gains from mass treatment, does not likely reflect the capability of these areas to support higher transmission rates. Remaining areas of higher prevalence here may serve as nearby sources of importation, yet the extent to which people move between these areas, the impact routine travel has on the risk of infection, or the contribution of this movement to the remaining malaria transmission in lower prevalence areas is unclear. Efforts assessing the infection prevalence associated with various types of travel and quantifying population mobility within this region would provide insights into the role movement plays with respect to current elimination efforts. In addition, in the context of MDA, understanding the degree of mobility in this setting would supplement discussion of intervention impact, as higher rates of travel outside the study region would reduce the epidemiological coverage of treatments.

To this end, here we examine associations between human movement and malaria parasite prevalence in the Lake Kariba region, an area targeted for elimination in the next few years. Specifically, this analysis uses data collected during household visits during the trial to 1) quantify the proportion of the population that had recently traveled, 2) assess the relationship between recent travel and individual *Plasmodium falciparum* malaria infection and identify characteristics of travel that modify that relationship, and 3) report the proportion of infections with a recent travel history. In addition, this analysis uses data from four time points over 2 years to examine seasonal differences in short-term travel rates and differences in the relationship between infection and travel, as malaria prevalence declined throughout the region.

## METHODS

Four MDA rounds with dihydroartemisinin–piperaquine, coinciding with the low (two rounds) (June–December)- and high (two rounds) (January–May)-transmission seasons, were conducted each year for two consecutive years in Southern Province, Zambia, during December 2014, February 2015, October 2015, and February 2016 as part of a community randomized controlled trial. The aims and methods of the trial are discussed in detail elsewhere.^5^ In brief, the trial was conducted within 60 geographically delineated HFCAs covering approximately 15,800 km^2^ of the Lake Kariba region of Southern Province. Forty of the included HFCAs were randomly allocated (20 per arm) to receive one of two MDA treatment modalities. For each round of MDA conducted, field teams attempted to visit all households within these HFCAs.

During household visits, all present household members aged 3 months or older were tested for *P. falciparum* malaria using histidine-rich protein 2 (HRP2) malaria rapid diagnostic tests (RDTs) (Standard Diagnostics Inc., Gyeonggi-do, Republic of Korea), provided treatment as per the study protocol, and administered questionnaires on environmental and behavioral risk factors for malaria, including history of indoor residual spraying (IRS), long-lasting insecticide-treated net (LLIN) ownership and usage, and recent travel. Household members, or a representative, reported whether individuals had spent at least one evening away from their place of residence within the previous 2 weeks, including the trip destination, the total nights away, and whether during travel, an LLIN was used while sleeping. If a trip was within the study area of Southern Province, the destination HFCA was noted by the interviewer; otherwise, the destination district or country was reported.

**Figure 1.**
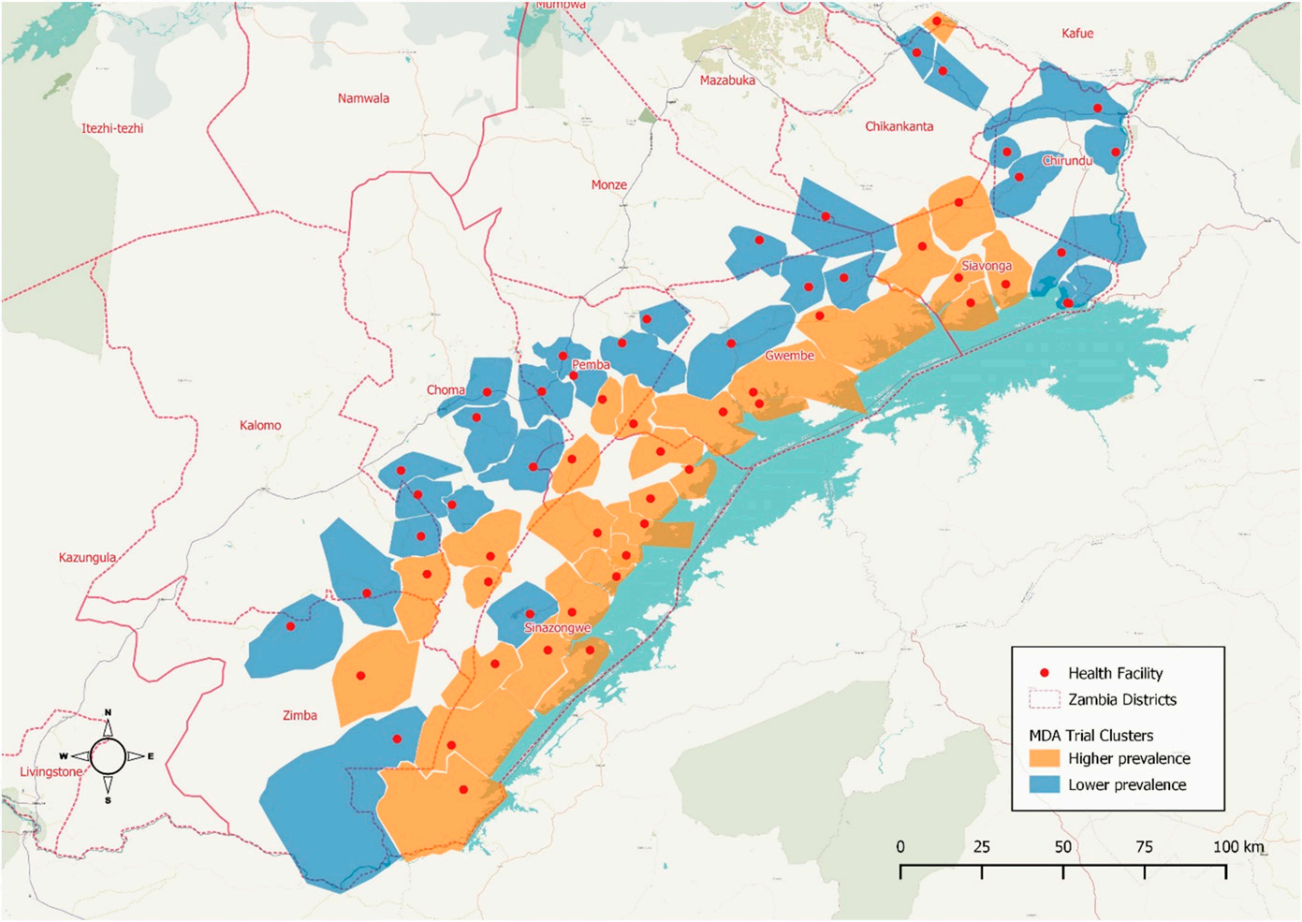
Malaria transmission levels in Southern Province health facility catchment areas (HFCAs), April/May 2014. This figure appears in color at www.ajtmh.org.

Travel status was categorized dichotomously as any or no reported trip within the previous 2 weeks. Using *P. falciparum* prevalence (*Pf*PR) measured as the proportion of RDT-positive children aged 1–59 months during a baseline cross-sectional survey in 2014, HFCAs were stratified into higher (> 10% parasite prevalence; median = 47.8%) or lower (< 10% parasite prevalence; median = 6.0%) transmission groups as part of the trial design (Figure 1). If travelers reported a destination within the trial area, these HFCA prevalence designations were used to classify each trip based on the level of prevalence at trip origin (residence) and destination HFCA into categories: lower to lower, lower to higher, higher to lower, or higher to higher. Data were not available to comparatively classify malaria prevalence in areas outside the study area.

All residents within HFCAs receiving either version of MDA—approximately 235,000 individuals in 37,000 households—were considered eligible for inclusion in this study. Consenting/assenting individuals visited during the trial with a valid RDT result and responding to questions on recent travel history were included in analysis. Data analysis was conducted in R version 3.5.1 (R Foundation for Statistical Computing, Vienna, Austria).^27^ Missing covariate data were imputed using the “mice” package for R.^28^ Chi-squared tests were used to compare the distribution of participant characteristics by travel status. Mixed-effects logistic regression models were used to estimate associations between RDT positivity and any reported travel, travel duration, LLIN use while traveling, and direction of travel with respect to origin and destination *Pf*PR (e.g., high-to-low prevalence and high-to-high prevalence). Adjusted odds ratios (AORs) were calculated controlling for participant age, gender, LLIN usage at home the previous evening, IRS at residence, trial arm, campaign round, and HFCA prevalence strata, including a random effect for the HFCA. Additional logistic models were used to estimate associations between travel and infection for each campaign round to assess differences in the relationship between infection and travel during various seasons, as parasite prevalence diminished throughout the study area with each subsequent round.

## RESULTS

In total, 680,045 line listings of household members were produced during household visits across four mass treatment rounds, and 94.8% were present and consented to participate in malaria testing. Valid RDT results were recorded for 602,171 consenting participants (93.4%). Recent travel history was further available for 602,294 (99.7%) participants with a valid RDT result.

Across the study area and all time points, 4,002 (0.7%) participants reported spending at least one evening away from home during the 2-week period before household visits. Compared with those reporting no travel, travelers were more likely older, male, to have slept under a net the previous evening at their place of residence, and to reside in a house that had not received IRS within the previous year (Table 1). A smaller proportion of individuals residing in higher prevalence areas reported any travel compared with those residing in lower prevalence areas (0.5% versus 0.8%; χ^2^ *P* < 0.001). The proportion of individuals traveling varied by HFCA but was generally greater in lower prevalence areas (median: 0.6%; interquartile range [IQR]: 0.4–1.2%) than that in higher prevalence areas (median: 0.4%; IQR: 0.2–0.7%). Distributions of traveler characteristics were similar among residents from lower and higher prevalence areas except that the proportion of female travelers was slightly lower in higher prevalence HFCAs (50.3% versus 53.5%; χ^2^ *P* = 0.050).

**Table 1:** Comparison of recent travelers and non-travelers during four mass drug administration rounds in Southern Province, Zambia, December 2014–March 2016

Characteristic	Traveled within the previous 2 weeks								
	All study participants			Residents of lower *Pf*PR areas*			Residents of higher *Pf*PR areas*		
	No	Yes	*P*-value (χ^2^)	No	Yes	*P*-value (χ^2^)	No	Yes	*P*-value (χ^2^)
Female (column %)	54.2	52.3	< 0.001	54.4	53.5	0.002	54.0	50.3	< 0.001
Age category (column %) (years)	–	–	< 0.001	–	–	< 0.001	–	–	< 0.001
< 5	18.6	7.9	–	18.0	7.6	–	19.2	8.3	–
5–15	32.4	10.1	–	32.5	9.7	–	32.3	10.6	–
> 15	49.0	82.1	–	49.5	82.7	–	48.5	81.1	–
Slept under bed net the previous evening (column %)	40.0	51.6	< 0.001	41.8	51.3	< 0.001	38.1	52.1	< 0.001
House received indoor residual spraying within past 12 months (column %)	25.0	21.5	< 0.001	25.0	20.3	< 0.001	24.9	23.2	0.200
Total, *N* (row %)	596,292 (99.3)	4,002 (0.7)	–	300,523 (99.2)	2,409 (0.8)	–	295,769 (99.5)	1,593 (0.5)	–

*Pf*PR = *Plasmodium falciparum* prevalence.

* Higher and lower prevalence designations based on a *P. falciparum* prevalence cutoff of approximately 10% among children younger than 5 years.

The overall prevalence of *P. falciparum* infection by RDT was 5.2% across all rounds and HFCAs. Among those positive for malaria, 1.4% had reported recent travel. Infection prevalence was higher among travelers than those among non-travelers—11.0% versus 5.1% (χ^2^ *P* < 0.001), respectively (Table 2). Adjusting for potential confounding factors, the odds of malaria infection among those traveling was 2.55 times higher (95% CI: 2.28–2.85, *P* < 0.001) than those who had not traveled recently. The number of days spent traveling was also positively associated with infection. The magnitude of this association was even greater for those traveling for longer periods to higher prevalence areas. Stratifying by lower/higher HFCA prevalence, a greater proportion of prevalent infections from lower prevalence areas had a recent history of travel—4.0% versus 0.9% (χ^2^ *P* < 0.001). Any travel was associated with increased odds of infection in both strata, although time spent away was not associated with an increase in the odds of infection among those from higher prevalence areas.

**Table 2:** Malaria prevalence and adjusted odds of RDT positivity among travelers, stratified by transmission intensity at residence

	All study participants			Residents of lower transmission areas			Residents of higher transmission areas		
Type of travel	*N*	% RDT (+)	AOR* (95% CI)	*N*	% RDT (+)	AOR* (95% CI)	*n*	% RDT (+)	AOR* (95% CI)
Travel within the previous 2 weeks									
None	596,292	5.1	Ref.	300,523	1.6	Ref.	295,769	8.7	Ref.
Any	4,002	11.0	2.55† (2.28–2.85)	2,409	8.5	5.18† (4.42–6.07)	1,593	14.9	1.62† (1.39–1.87)
Number of days traveled									
1–5	2,532	9.8	Ref.	1,536	7.6	Ref.	996	13.2	Ref.
6–10	637	12.7	1.46† (1.10–1.95)	396	10.4	1.72 (1.13–2.62)	241	16.6	1.31 (0.88–1.95)
11+	697	14.3	1.63† (1.24–2.15)	412	10.7	2.00† (1.31–3.03)	285	19.6	1.42 (0.99–2.05)
Traveling (days) by destination, area prevalence									
1–5, lower	912	8.3	Ref.	693	7.1	Ref.	219	12.3	Ref.
6–10, lower	182	9.3	1.07 (0.60–1.93)	134	7.5	1.03 (0.47–2.27)	48	14.6	1.19 (0.47–2.96)
11+, lower	210	9.5	1.07 (0.62–1.85)	131	6.1	1.01 (0.43–2.33)	79	15.2	1.18 (0.55–2.50)
1–5, higher	697	14.8	1.19 (0.84–1.70)	190	15.3	1.07 (0.59–1.93)	507	14.6	1.08 (0.67–1.76)
6–10, higher	155	22.6	2.22† (1.36–3.61)	43	25.6	3.96† (1.69–9.30)	112	21.4	1.66 (0.89–3.10)
11+, higher	137	24.1	2.09† (1.26–3.46)	30	10.0	1.19 (0.32–4.40)	107	28.0	1.99 (1.08–3.67)
1–5, unknown	923	7.5	0.80 (0.55–1.15)	653	6.0	0.72 (0.44–1.17)	270	11.1	0.88 (0.49–1.55)
6–10, unknown	300	9.7	1.20 (0.75–1.94)	219	9.1	1.39 (0.77–2.52)	81	11.1	0.93 (0.41–2.10)
11+, unknown	350	13.4	1.76† (1.16–2.68)	251	13.1	2.23† (1.31–3.80)	99	14.1	1.07 (0.52–2.19)
Bed net use while traveling									
Did not use bed net	2,519	12.1	Ref.	1,500	9.8	Ref.	1,019	15.6	Ref.
Used bed net	1,429	9.2	0.74† (0.63–0.87)	873	6.2	0.55† (0.42–0.72)	556	14.0	0.93 (0.75–1.16)

AOR = Adjusted odds ratios; RDT = rapid diagnostic test; *Pf*PR = *Plasmodium falciparum* prevalence.

* Adjusted odds ratio: adjusted for gender, age, indoor residual spraying and long-lasting insecticide-treated net usage at home, time period, campaign intervention type, and malaria prevalence in the area of residence.

† *P* < 0.01.

Throughout this population and across time points, a greater proportion of individuals reported travel to lower prevalence (33.3%) compared with higher prevalence areas (25.4%; Table 3). The remaining proportion of travelers either reported a destination outside the trial area or did not report a destination (15.2% and 26.1% of all travelers, respectively). Among those reporting travel outside the trial areas, approximately one-third of these trips were to nearby lower prevalence urban areas such as Lusaka and Livingstone districts. Only 10 individuals reported travel outside Zambia. Although infections among those traveling to higher prevalence areas were twice as common, the odds of infection for these groups was similar after accounting for individual, household, and home HFCA factors.

**Table 3:** Travel rates by transmission intensity at traveler residence and destination

*Pf*PR at residence	*Plasmodium falciparum* prevalence. at destination	*N*	% Of population	% Of all travelers	% Rapid diagnostic test(+)	% Used bed net while traveling	AOR* (95% CI)
Any (*N* = 600,294)	None	596,292	99.33	–	5.1	–	Ref.
	Lower	1,332	0.22	33.3	8.6	37.9	2.65† (2.15–3.26)
	Higher	1,016	0.17	25.4	17.3	36.1	2.23† (1.87–2.67)
	Unknown	1,654	0.28	41.3	9.1	34.9	2.88† (2.39–3.47)
	Any	4,002	0.67	100.0	11.0	36.2	2.55† (2.28–2.85)
Lower (*N* = 302,932)	None	300,523	99.20	–	1.6	–	Ref.
	Lower	978	0.32	40.6	6.9	36.9	3.96† (3.05–5.14)
	Higher	267	0.09	11.1	16.1	47.6	7.38† (5.11–10.67)
	Unknown	1,164	0.38	48.3	8.1	34.1	5.76† (4.59–7.22)
	Any	2,409	0.80	100.0	8.5	36.8	5.18† (4.42–6.07)
Higher (*N* = 297,362)	None	295,769	99.46	–	8.7	–	Ref.
	Lower	354	0.12	22.2	13.3	40.7	1.66† (1.20–2.29)
	Higher	749	0.25	47.0	17.8	31.9	1.76† (1.44–2.15)
	Unknown	490	0.16	30.8	11.6	36.6	1.35 (1.01–1.80)
	Any	1,593	0.54	100.0	14.9	35.3	1.62† (1.39–1.87)

AOR = adjusted odds ratios.

* Adjusted odds ratio: adjusted for gender, age, indoor residual spraying and long-lasting insecticide-treated net usage at home, time period, and campaign intervention type.

† *P* < 0.01.

Analysis stratified by traveler origin further parsed travel by the prevalence of both trip origin and destination and revealed differences in travel rates and malaria infection among subgroups (Table 3). Individuals were more likely to travel to HFCAs with similar malaria prevalence, largely explained by high intra-HFCA travel rates. Those traveling from higher to higher prevalence HFCAs experienced the greatest proportion of infections of any subgroup (17.8%), although travel from HFCAs of higher prevalence was only associated with a relatively moderate increase in the odds of infection, regardless of destination. The greatest odds of infection was observed among those traveling from lower prevalence areas—whether to areas of similarly lower prevalence (AOR: 3.96) or areas of higher prevalence (AOR: 7.38).

Rates of travel also differed by season and year (Table 4), with higher rates reported during the early rainy and dry seasons (December 2014 and October 2015, respectively) than mid-to-late rainy seasons (February 2015 and February 2016). Although fewer individuals were tested and interviewed during rainy seasons, the seasonal differences in participants corresponded to the relative proportion of households visited during each time points as indicated by similar numbers of participants per household during each round (Table 4), that is, the reduction in participants during rainy seasons was likely because of fewer household visits rather than differences in absence at the time of each visit. The largest proportion of participants reported travel in the 2 weeks preceding the mass treatment round in December 2014, possibly related to end-of-year holiday travel. The study area experienced an overall decrease in malaria infections throughout the study period; malaria prevalence was 8.5%, 4.8%, 5.4%, and 1.7% in December 2014, February 2015, October 2015, and February 2016 campaign rounds, respectively. Prevalence was consistently higher among travelers than that among non-travelers at each time point. The adjusted odds of infection were greatest among travelers during the transmission seasons in both 2015 and 2016, although the proportion of infections with a travel history did not significantly differ across time points.

**Table 4:** Seasonal differences in travel and adjusted odds of RDT positivity

Mass drug administration round	Households visited	Participants		Traveled			Used bed net while traveling		RDT (+)		
		*N*	*N* per household	Yes/no	*N*	Column %	*N*	Row %	*N*	Row %	AOR* (95% CI)
Round 1 (December 2014)	36,201	161,268	4.5	No	159,279	98.8	–	–	132,65	8.3	Ref.
				Yes	1989	1.2	661	33.7	205	10.3	2.20† (1.87–2.59)
Round 2 (February 2015)	29,344	125,904	4.3	No	125,406	99.6	–	–	5,949	4.7	Ref.
				Yes	498	0.4	221	45.4	67	13.5	3.50† (2.64–4.63)
Round 3 (October 2015)	36,050	164,390	4.6	No	163,350	99.4	–	–	8,754	5.4	Ref.
				Yes	1,040	0.6	339	32.9	128	12.3	2.40† (1.94–2.97)
Round 4 (February 2016)	31,525	148,732	4.7	No	148,257	99.7	–	–	2,512	1.7	Ref.
				Yes	475	0.3	208	44.3	41	8.6	6.67† (4.68–9.51)

AOR = adjusted odds ratios; RDT = rapid diagnostic test.

* Adjusted odds ratio: adjusted for gender, age, indoor residual spraying and long-lasting insecticide-treated net usage at home, time period, campaign intervention type, and malaria prevalence in the area of residence.

† *P* < 0.01.

Among participants spending at least one evening away from home, 98.6% also reported on whether an LLIN was used while traveling. In total, 36.2% stated that a net had been used at least some evenings during the time away. Those sleeping under a net at home, aged between 5 and 15 years, and traveling from lower to higher prevalence areas were significantly more likely to use a net also while away. Net usage while traveling differed by season, reported at 45.4% and 44.3% during high-transmission seasons (rounds 2 and 4) compared with 33.7% and 32.9% during low-transmission seasons (rounds 1 and 3), respectively (Table 4). Malaria prevalence and the adjusted odds of infection were lower for those sleeping under an LLIN while away (9.2% versus 12.2%), and the adjusted odds of malaria infection were 26% lower among participants using an LLIN while traveling (Table 2). Among traveler subgroups, net use was highest among participants traveling to areas of different prevalence compared with their area of residence (Table 3), either from lower to higher areas (47.6%) or higher to lower areas (40.7%).

## DISCUSSION

Using survey data collected during four rounds of a mass treatment campaign spanning two consecutive years, we assessed recent short-term travel rates among residents and the relationship between this travel and individual *P. falciparum* infection status in a region of heterogeneous malaria transmission. Results demonstrated a clear association between recent overnight travel and malaria infection. Although malaria prevalence was estimated to be 5.1% over all MDA rounds, prevalence among those traveling within 2 weeks before testing was more than twice this amount. Malaria infections were even more likely among those taking trips of longer duration, especially to higher prevalence areas.

The magnitude of the association between travel and infection differed by transmission levels at the origin and destination of travel, as well as seasonal differences in transmission. Among individuals from lower transmission areas, travel to areas of higher transmission was associated with a substantial increase in infections. Although travel was still associated with infection among individuals already living in higher transmission areas, destination did not seem to make a difference in the prevalence of infection likely because of the already higher probability of acquiring an infection within these areas. Seasonal differences in the travel–infection relationship corresponded to annual fluctuations in malaria transmission seen in this region (i.e., increased odds of malaria infection among travelers during rainy seasons).

Only a small proportion of residents reported recent travel (from 0.3% to 1.2% across campaign rounds), resulting in relatively few prevalent infections with a recent history of travel throughout this population. Travel rates were slightly higher among those residing in lower prevalence areas possibly because of geographic differences between lower and higher prevalence catchment areas. Lower prevalence areas are closer to major thoroughfares connecting urban areas (e.g., Lusaka, Choma, and Livingstone), whereas higher prevalence areas tend to be closer to Lake Kariba, where more challenging topography and poorer quality roads make travel more difficult. Observing human mobility within this same population, Searle et al.^29^ described seasonal differences in mobility among rural residents and reported that the rate of long-distance travel was noticeably reduced during rainy seasons. Here, we observed a similar pattern in travel, with a reduction in travel during high-transmission seasons believed to be due to changes in rural road conditions. Currently, reductions in travel likely serve to mitigate expected increases in the prevalence of infection attributable to travel during higher transmission seasons, but this effect may wane with future improvements in transportation infrastructure in rural areas.

Throughout the 2-year period of this study, the season-specific proportion of infections related to travel increased with decreasing prevalence, suggesting that the proportional role of human movement in malaria infection may become more important as malaria prevalence further declines. Yet, widely implemented strategies for malaria control, such as LLIN distribution and promotion, may significantly reduce the role of regional movement in achieving and maintaining malaria-free areas. Net usage while traveling was associated with a significant reduction in the odds of malaria infection. Although overall net usage while traveling did not differ between residents of lower and higher transmission areas, higher rates were observed among subgroups traveling away from their area of residence, suggesting that differences in perceived risk may affect adoption of preventative measures. This is further supported by an increase in net usage while traveling during the peak transmission seasons of 2015 and 2016.

It should be noted that the general limitations of cross-sectional designs used in other assessments of the impact of individual travel on malaria infection apply to this study as well. While estimating the strength of associations between travel and infection prevalence, the design of this study did not allow for further insight into the causal relationship between malaria and travel. Although travel has been recognized as a risk factor for malaria generally and in sub-Saharan Africa, this study did not allow us to identify the timing of infection with regard to travel—whether infection occurred before, during, or after travel, or whether travel was specifically to seek care for a symptomatic infection. The recall period used in this study was somewhat short at 2 weeks and likely led to both autochthonous infections being misclassified because of travel and vice versa. Infections acquired locally before leaving the area would have been considered as travel related in analysis, especially for very recent overnight trips. Infections acquired while traveling outside in the 2-week recall period would have been incorrectly classified as locally acquired. Extending the recall window would have addressed the latter issue but may have increased the probability of individuals seeking and receiving treatment before household visits, leading to some travel-related infections to appear negative by RDTs. Also, a longer recall period would have increased the probability of incorrectly classifying infections acquired after travel. It is likely that both types of misclassification occurred, although the resultant net change on effect estimates (i.e., bias toward or away from the null association) is unclear.

Single household visits provided snapshots of population travel rates but were limited in producing a comprehensive summary of circulation within this population needed to fully assess the role of movement in infections. Two-week travel rates do not indicate the frequency of travel or cumulative travel time throughout the year—a key dimension of the risk for parasite acquisition and thus importation. In addition, travel histories would have omitted those away at the time of surveys, underestimating the proportion of population traveling and, by extension, estimates of the proportion of infections attributable to travel. Timing of rounds may also have captured momentary peaks or troughs in travel rates not reflective of travel for the full season, although the duration of each round across the study area likely improved representativeness of these estimates.

Finally, this study considered only travel among those residing in the study area. Presumably, individuals from other malaria-endemic provinces and countries travel to this area and may also contribute to local transmission. Unless visitors comprise the vast majority of travelers in the area, they likely contribute less to importation than returning residents.^14^ Nevertheless, their role may be significant to the long-term success of elimination efforts^11^ and should be included in a more robust assessment of the influence of human movement on continuing transmission.

Conducting household surveys in the context of mass malaria testing afforded a rare opportunity for examining the prevalence of human movement and its association with malaria infection throughout a population. Questionnaires provided information on household and individual risk factors—including travel destination, IRS exposure, LLIN usage, age, etc.—for an estimated 80.7% of households among a population of approximately 235,000.^30^ Previous studies on this topic have often been health facility based, using a case–control design to compare travel exposure and infection among patients.^19–24^ That design, although providing insights into the role of travel on individual malaria infection, does not lend itself to estimates of the relative importance of importation to current malaria prevalence and has often been limited in its ability to consider differences in risk due to characteristics of travel. Not all travel impose a substantial risk to malaria infection, and consideration of risk at trip destination is important; movement between areas with little transmission or during times with low vector activity is unlikely to have a substantial impact on travelers and onward transmission compared with travel to highly endemic areas.^25^ Including low-risk types of travel in estimations of the travel impact may understate the importance of some types of travel, indicating a need for better characterization of travel to adequately assess the impact of human movement and identify those most at risk, as previously suggested by Smith and Whittaker.^25^ Large-scale population-wide data collection used in this study provided a sufficient sample size to examine differences in categories of travel, such as travel direction, duration, distance, and season. Repeated data collection within the same population also provided insights into the changing role of travel in malaria infections during a period of diminishing prevalence.

Southern Province, Zambia, has seen substantial reductions in overall malaria infection prevalence within the past few years,^1^ and continued efforts of the national program and partners promise to push this even lower. Results from this analysis coincide with conclusions from others that short-term travel can pose a substantial risk of malaria infection and further suggest that this risk increases as local malaria prevalence declines. Although the proportion of infections directly associated with recent travel was relatively low, the threat of importation is likely to be increasingly important for achieving elimination regionally as prevalence declines. Moving forward, the national program will likely benefit from continued expansion of control and elimination efforts into surrounding provinces to address sources of transmission connected by movement. Last, the use of bed nets while traveling is promising, as it suggests that residents are risk conscious and receptive to adopting preventative measures even while outside their primary residence. Continued promotion of existing malaria prevention methods such as LLIN usage and/or targeting at-risk individuals with chemoprophylaxis may prove important for establishing and maintaining malaria-free areas in the long term.
